# Self-esteem mediates the relationship between physical activity and smartphone addiction of Chinese college students: a cross-sectional study

**DOI:** 10.3389/fpsyg.2023.1256743

**Published:** 2024-01-05

**Authors:** Yuanyuan Ke, Xiuxia Liu, Xue Xu, Bingchen He, Jinfu Wang, Lijun Zuo, Haiyun Wang, Guan Yang

**Affiliations:** ^1^School of Physical Education, South China University of Technology, Guangzhou, Guangdong, China; ^2^Department of Physical Education, Xiamen University, Xiamen, Fujian, China; ^3^School of Finance and Economy, Guangdong Engineering Polytechnic, Guangzhou, Guangdong, China; ^4^School of Physical Education, Guangzhou College of Commerce, Guangzhou, Guangdong, China

**Keywords:** behavioral addiction, smartphone addiction, physical activity, self-esteem, mediating effect, college students, a cross-sectional study

## Abstract

**Objective:**

Smartphone addiction, as a key topic in the current field of behavioral addictions and public health, has brought many negative impacts on the physical, psychological, interpersonal communication, and even academic performance among contemporary college students. Therefore, the purpose of this study is to offer ideas for solving smartphone addiction among college students through investigating the potential mediating effect of self-esteem in the relationship between physical activity and smartphone addiction.

**Methods:**

By the quota sampling, a cross-sectional study was conducted to investigate 650 college students from 10 colleges in Guangzhou Higher Mega Center, and several self-reported instruments including physical activity rating scale-3 (PARS-3), mobile phone addiction tendency scale (MPATS), self-esteem scale (SES) were used to collect the related data needed for the present study. The descriptive analysis, correlation analysis, hierarchical regression analysis, and mediating effect analysis in this study were performed in turn.

**Results:**

The results showed that physical activity (*r* = −0.124, *p* < 0.01) and self-esteem (*r* = −0.360, *p* < 0.01) were all negatively correlated with smartphone addiction, and both could also significantly and negatively predict smartphone addiction. There was a positive correlation between physical activity and self-esteem (*r* = 0.084, *p* < 0.05), and self-esteem could be significantly predicted by physical activity. And more important, the relationship between physical activity and smartphone addiction could be partially mediated by self-esteem, and the indirect effect value was −0.346 (95% Boot CI = −0.695; −0.023), along with the mediating effect accounted for 24% of the total effect between physical activity and smartphone addiction.

**Conclusion:**

The current study shows that physical activity could not only directly reduce smartphone addiction, but also decrease smartphone addiction by indirectly improving self-esteem, which is important in practice for solving this troublesome issue and then gradually developing a healthy behavior in daily life for college students in China, and even across the world in near future.

## 1 Introduction

As an indispensable and practicable tool, smartphones have gradually penetrated into various aspects of people’s daily life, such as social interaction, entertainment, office work, shopping, and wealth management (Gökçearslan et al., 2016). However, while smartphones bring considerable conveniences to human beings, the problem of smartphone addiction caused by excessive usage or pathological usage with smartphones has been also paid much attention for us (Yang et al., 2021). Nowadays, smartphone addiction is ubiquitously used among citizens, especially the population of college students (Jun, 2016; Chen et al., 2022). The previous research shows that the rate of smartphone addiction among teenagers has exceeded 25% in China (Tang et al., 2016), which is higher than that in global youth with 23.3% (Sohn et al., 2019), and likewise, the prevalence of mobile phone addiction in the group of college students ranges from 21.4 to 27.4% (Jun, 2016). As we all know, smartphone addiction not only leads to poor academic performance and sleep quality among college students (Li et al., 2015; Hawi and Samaha, 2016; Rathakrishnan et al., 2021; Kao, 2023), but also brings a series of mental health problems to them, such as anxiety, depression, loneliness, as well as the poor interpersonal relationships (Jun, 2016; Kim and Kim, 2016; Wang et al., 2017; Diotaiuti et al., 2021; Ou-Yang et al., 2022). It is admittedly that owing to the serious negative effect from the COVID-19 Lockdown in recent years, the issue that college students’ psychological adjustment and reaction during facing this emergent incident has been greatly attracted a large number of attention by researchers at alien and domestic now (Diotaiuti et al., 2021), and in particular, those individuals who have largely addicted themselves to the these advanced digital communicating devices, such as the computers and smartphones (Diotaiuti et al., 2022a,b).

Obviously, due to smartphone addiction has caused serious problems on the physical and mental health for college students, and therefore, how to prevent it or solve it has become an important challenge for us now. Fortunately, some preceding studies have disclosed that a significant and negative correlation was between physical activity and smartphone addiction (Choi, 2015; Kim et al., 2015; Yang et al., 2019; Lin et al., 2022). physical activity, as a manner of physical exercise with a certain intensity, frequency, and duration (Liu, 2020), can not only improve physical fitness, but also greatly contribute to mental health (Ho et al., 2015). According to the theory of temporal self-regulation of physical activity (Salmon, 2001; Chen et al., 2022), many behaviors beneficial for the individual body are usually generated by regular physical activity, and it can be easily seen that reasonably increasing physical activity plays an important role in personal health. Yang et al. (2021) pointed out that the higher level of physical activity means the lower probability of smartphone addiction for college students in their daily life; and the author also put forth that moderate-intensity acute aerobic exercise could reduce college students’ desire for mobile phones (Yang et al., 2022). That is, physical activity could reduce the probability of addiction to smartphones in some degree. Based on these persuasive evidence above, it is not difficult to detect that there is a negative correlation between physical activity and smartphone addiction, which means actively engaging in physical activity in daily life could effectively reduce the possibility of addicting to smartphones.

Self-esteem, as an individual’s evaluation of their self-worth and a positive or negative attitude toward themselves (Baumeister et al., 2003), has been closely related to mental health for human beings (Gao et al., 2015; Ding et al., 2020). As one of the valid assessment indicators for mental health, self-esteem has a very significant impact on the comprehensive development among college students. However, the fear management theory of self-esteem points out that when the persons encounter adverse environments that are not conducive to the development of self-esteem, they would use some specific methods to self-compensate; specifically speaking, the greater threat from self-esteem for them, the more likely to engage in problematic or abnormal behavior to compensate themselves, including smartphone dependency (Yang et al., 2004; He et al., 2020). In other words, when college students’ self-esteem may not be satisfied in time, they probably use mobile phones to compensate themselves, which ultimately leads to a severe issue, namely smartphone addiction. Consistent with the published research, individuals with low self-esteem would be more likely to develop smartphone addiction (Sung, 2014; He et al., 2019; Zeidan et al., 2021). Undoubtedly, based on these studies above, it can be easily proposed that self-esteem is negatively correlated with smartphone addiction in a large degree.

As an essential topic in the field of sports and exercise psychology, self-esteem has frequently attracted widespread attention among researchers. Similarly, as an effective way to reduce fatigue and relieve stress, physical activity has been always playing an important role in personal mental health. To date, several prior work has explored the potential relation between physical activity and self-esteem. Zhang puts forth that when teenagers faced with considerable academic pressure, their normal interpersonal relationships would be largely disturbed, but the physical activity can effectively alleviate this issue (Zhang, 2016). Simultaneously, actively engaging in physical activity can further enhance physical self-esteem level (Chen, 2012), and then the overall sense of wellbeing for college students could be obviously improved (Eather et al., 2016; Sun and Zhang, 2020). Moreover, several work has suggested that frequently participating in extracurricular exercise can effectively enhance self-esteem level (Yìğìter, 2014; Zhang et al., 2017); given that, as for college students, the higher self-esteem level they hold, the healthier mental health will be possessed by them (Xu et al., 2020). Additionally, in accordance with the exercise self-esteem model, physical activity could improve physical self-worth such as motor function, physical fitness, and physical condition, and then further enhance people’s self-esteem (Sonstroem and Morgan, 1989). Based on those research mentioned above, it could be reasonably suggested that actively participating in physical activity could effectively improve the self-esteem among college students.

From these related literature review discussed above, it is not difficult to find that smartphone addiction caused by inappropriate or incorrect use of mobile phones would bring lots of passive effects to college students, and oppositely, as a positive and beneficial lifestyle, physical activity could effectively resolve this troublesome issue by indirectly improve individuals’ self-esteem. In accordance with the interaction of person-affect-cognition-execution (I-PACE) model, addictive behavior such as smartphone addiction, is the result of a combination of triggering factors, mediators, and methods of execution (Brand et al., 2016; Li et al., 2022). Hence, it can be recognized that the low-level physical activity may be the triggering factors for smartphone addiction, and at the same time, that the improvement of self-esteem level by the enhancement of physical activity may play the mediating role in reducing the smartphone addiction. Given that, the present study hypothesized that physical activity would decrease smartphone addiction by indirectly improving self-esteem among college students; that is, self-esteem possibly mediates the relationship between physical activity and smartphone addiction.

## 2 Materials and methods

### 2.1 Procedures and participants

This study was carried out in accordance with the 1964 Declaration of Helsinki and its later amendments or comparable ethical standards, and also reviewed and approved by the Ethics Committee of South China University of Technology. This study used a cross-sectional survey to collect relevant data, and all of the data were gathered using several self-reported standard scales. The data collected in this study followed the principle of confidentiality, and all research subjects were totally voluntary. Before the formal survey, all participants gave written informed consent to us, at the same time, they were also informed to quit this survey at any time. Through the quota sampling, 650 college students were chosen from 10 colleges in Guangzhou Higher Mega Center, China, and 65 college students was conveniently selected from each college. If the participant usually communicates with other people by smartphone in daily life, and without any inborn diseases both from physical or mental aspects, he or she would be an eligible candidate to engage in this study. Among these, however, 42 samples were excluded due to incorrect or incomplete data, such as several missing or blank items in their answers, and he or she did not use smartphones in theirs daily life up till now, and even unfortunately someone had congenital motor dysfunction. Therefore, the final sample sizes in this study were 608, and the response rate of the questionnaire in the current article was 93.53%, which consists of 320 female (52.63%) and 288 male (47.37%) respondents. The sample included 317 (63.9%) liberal arts students and 291 (36.1%) science students. Furthermore, 253 college students (41.61%) were from rural area, and 355 participants (58.39%) from urban area, along with the average age of them was (20.27 ± 1.69) years.

### 2.2 Measures

#### 2.2.1 Physical activity rating scale-3

Physical activity, was measured using the physical activity rating scale-3 (PARS-3) created originally by Liang and Liu (Liang and Liu, 1994), which includes three aspects, namely exercise duration, exercise frequency, and exercise intensity. The score range for each item is from 1 to 5, and the calculation formula of total score is intensity × (time-1) × frequency, with a range from 0 to 100. According to previous articles (Xia et al., 2018), the amount of physical activity can be divided into three levels from 1 (light exercise) to 3 (vigorous exercise): namely light exercise, moderate exercise and vigorous exercise. Light exercise is less than or equal to 19 points, moderate exercise is defined as 20 to 42 points, and equal to or more than 43 points would be classified as vigorous exercise. The Cronbach’s α of PARS-3 in the current research was 0.693, which is basically satisfactory.

#### 2.2.2 Self-esteem scale

Self-esteem, was measured using the self-esteem scale (SES) revised by Ji and Yu (1999) in order to objectively assess the overall self-esteem level of college students in China. The scale was compiled by Rosenberg (1965), and it was also a single dimensional scale consisting of ten items. The total score ranges from 4 to 40, and the higher score means the higher self-esteem level. Among them, items 3, 5, 8, 9, and 10 were reverse scoring questions. However, according to existing research (Tian, 2006; Shen and Cai, 2008; Wang et al., 2010), it was found that there were differences in cultural context when the scale was revised, so the item 8 would be conducted by a positive scoring manner in Chinese cultural context. And the SES has a good confidence level in this article, due to the Cronbach’s α itself was 0.826.

#### 2.2.3 Mobile phone addiction tendency scale

Smartphone addiction, was measured using the mobile phone addiction tendency scale (MPATS) developed by Xiong J. et al. (2012), which has been compiled based on Young’s internet addiction scale (Young, 1998) and other relevant knowledge about the Internet addiction (Johansson and Götestam, 2004; Dowling and Quirk, 2009). It is a 5-point-Likert scale that includes 16 items and 4 dimensions: namely withdrawal symptom, salient behavior, social comfort, and mood change. The score for each item is from 0 (completely disagree) to 5 (completely agree), with the total score of 16 to 80 points. At the same time, higher score indicates a higher tendency addicted to mobile phones. The Cronbach’s α of the MPATS in this study was 0.894, which presents a perfect reliability.

### 2.3 Statistical analysis

All data in the present study were calculated using SPSS 26.0 software. Continuous variables with normal distribution were displayed using mean (M) ± standard deviation (SD), while categorical variables were presented using frequencies and percentages. Firstly, the Harman’s single-factor analysis method was used to ensure that the present research did not have obvious problem of common method variance. Secondly, the descriptive and correlation analysis were conducted to verify the relation between physical activity, self-esteem, and smartphone dependency. Thirdly, hierarchical regression analysis was used to test the predictive level of each variable on the dependent variable. Finally, model 4 from Process 4.0 in SPSS macro was utilized to analyze the mediating effect of self-esteem in the relationship between physical activity and smartphone dependency, and the bootstrapping method was also used to obtain 95% bias-corrected percentile confidence intervals (CI) generated by resampling data with 5,000 times (Hayes, 2013). The significance level of the this study was set at *P* < 0.05.

## 3 Results

### 3.1 Test for common method variance

The common method bias analysis was conducted through the Harman’s single-factor test in the present study. In accordance with the final analysis result, there were 7 factors with the original root greater than 1, and the first common factor could explain 26.61% of the cumulative variance, which is less than the critical value with 40% required by the corresponding standard (Podsakoff et al., 2003; Xiong H. X. et al., 2012). Hence, there was no serious problem of common method bias in this research.

### 3.2 Descriptive statistics and correlation analysis

The results of descriptive statistics and correlation analysis were shown in Table 1. As is shown below, the average score of college students’ physical activity amount was 17.61, which is located in the stage of the light-level exercise. Simultaneously, their self-reported score of smartphone addiction and self-esteem were all the moderate-level range, along with score of 42.81 and 28.52, respectively. In terms of correlation, physical activity was significantly and negatively correlated with smartphone addiction (*r* = −0.124, *p* < 0.01), but positively and significantly correlated with self-esteem (*r* = 0.084, *p* < 0.05). In addition, smartphone addiction was inversely associated with self-esteem (*r* = −0.360, *p* < 0.01).

**TABLE 1:** Descriptive statistics and correlation analysis for main variables.

Variable	M	SD	Physical activity	Smartphone addiction	Self-esteem
Physical activity	17.61	5.61	–		
Smartphone addiction	42.81	10.63	−0.124**	–	
Self-esteem	28.52	4.77	0.084*	−0.360**	–

M, mean; SD, standard deviation;

*p < 0.05;

**p < 0.01.

### 3.3 Hierarchical regression analysis

Table 2 shows the hierarchical regression analysis of the demographic variables, physical activity, and self-esteem to predict the degree of smartphone addiction. As is shown below, gender, major, and source were put in Block 1, then physical activity was added to Block 2, and self-esteem followed in Block 3. In Model 1, gender was a positive factor in predicting smartphone addiction (β = 0.089, *p* < 0.05), explaining 2.2% of the variability of smartphone addiction. The physical activity negatively and significantly predicted smartphone addiction (β = −1.00, *p* < 0.05), accounting for 0.8% of the variance of smartphone addiction in Model 2. In the final Model 3, source could positively predict smartphone addiction and also reached a significant level (β = 0.095, *p* < 0.05), and self-esteem could negatively and significantly predict smartphone addiction (β = −0.369, *p* < 0.001), explaining 13.2% of the variability of smartphone addiction. In this table, the percentage of *R*^2^ increased from 2.2 to 16.2%, and self-esteem could be a negative and significant predictor for smartphone addiction.

**TABLE 2:** Hierarchical regression analysis of demographic indicators, physical activity, and self-esteem for smartphone addiction.

	Variable	Beta (β)	*t*	Δ *R*^2^	Δ *F*	*R* ^2^	*F*
Model 1				0.022	4.427**	0.022	4.427**
Block 1	Sex	0.089	2.080*				
	Major	−0.075	−1.757				
	Source	0.042	1.021				
Model 2				0.008	5.224*	0.030	4.649**
Block 1	Sex	0.049	1.054				
	Major	−0.075	−1.753				
	Source	0.043	1.063				
Block 2	Physical activity	−1.00	−2.286*				
Model 3				0.132	94.526***	0.162	23.201***
Block 1	Sex	0.078	1.811				
	Major	−0.055	−1.388				
	Source	0.095	2.488*				
Block 2	Physical activity	−0.057	−1.385				
Block 3	Self-esteem	−0.369	−9.722***				

Beta (β), standardized coefficients; *R*^2^, *R* square; Δ*R*^2^, *R* square change; Δ*F*, *F* Change;

*p < 0.05;

**p < 0.01;

***p < 0.001.

### 3.4 Mediating effect analysis

**FIGURE 1:**
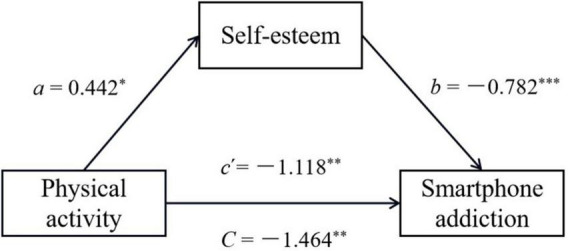
The path coefficient of mediating effect for self-esteem between physical activity and smartphone addiction. *C* = total effect; *a, b, c’* = direct effect; *p < 0.05; **p < 0.01; ***p < 0.001.

Table 3 shows the mediating effect of self-esteem between physical activity and smartphone addiction via stepwise regression analysis. Firstly, in the regression analysis of smartphone addiction on physical activity, physical activity was a negative predictor of smartphone addiction (β = −0.131, *p* < 0.01) and the model had a good fit [*F*_(1,606)_ = 10.643, *p* < 0.01]. At the same time, the 95% bootstrap confidence interval of the *B* excluded 0 and physical activity explained 1.7% of variance in smartphone addiction. In step 2, the regression analysis of self-esteem on physical activity revealed 0.8% of variance in self-esteem and the 95% bootstrap confidence interval excluded 0, which means physical activity could positively and significantly predict self-esteem (β = 0.088, *p* < 0.01). Likewise, this model also had an excellent fit [*F*_(1,606)_ = 4.773, *p* < 0.05]. Finally, the model of regression analysis for smartphone addiction on self-esteem and physical activity had a good fit [*F*_(2,605)_ = 10.643, *p* < 0.001] in step 3 and together explained 14% of the variance in smartphone addiction, along with both physical activity and self-esteem could negatively predict smartphone addiction (β = −0.351, *p* < 0.001; β = −0.100, *p* < 0.01). And as is clearly shown in Figure 1, self-esteem played a partial mediating role in the relationship between physical activity and smartphone addiction, and the indirect effect was −0.346 (95% Boot CI = −0.695; −0.023), which means physical activity could decrease smartphone addiction indirectly by improving self-esteem, and the mediating effect of self-esteem approximately accounted for 24% of the total effect.

**TABLE 3:** The mediating effect analysis of self-esteem between physical activity and smartphone addiction.

	*B*(SE)	Beta (β)	*t*	95% CI	*R*	*R* ^2^	*F*
Step 1					0.131	0.017	10.643 **
Physical activity → smartphone addiction	−1.464 (0.449)	−0.131	−3.262**	−2.346, −0.582			
Step 2					0.088	0.008	4.773*
Physical activity → self-esteem	0.442 (0.202)	0.088	2.184*	0.044, 0.839			
Step 3					0.374	0.140	49.050***
Self-esteem → smartphone addiction	−0.783 (0.084)	−0.351	−9.272***	−0.948, −0.616			
Physical activity → smartphone addiction	−1.119 (0.422)	−0.100	−2.650**	−1.947, −0.289			

B, unstandardized coefficient; SE, standard error; Beta (β), standardized coefficient; 95% CI, 95% bootstrap confidence intervals;

*p < 0.05;

**p < 0.01;

***p < 0.001.

## 4 Discussion

The present study was intended to disclose the underlying mediating mechanism between the relationship of physical activity and smartphone addiction, and ultimately found that physical activity could not only directly reduce smartphone addiction, but also indirectly decrease smartphone addiction by improving self-esteem. Taken this product into account, the hypothesis of the current study had been confirmed well. That is to say, self-esteem plays a mediating role between physical activity and smartphone addiction, and the mediating effect of self-esteem accounted for approximately 24% of the total effect between physical activity and smartphone addiction.

In recent years, as we all know, as an important research hotspot in the field of behavioral addiction and public health, smartphone addiction has increasingly attracted widespread attention for relevant researchers. Unreasonable or incorrect use of mobile phones not only leads to poor academic performance for college students (Hawi and Samaha, 2016), but will also lead to several latent risks such as decreased life satisfaction and sleep quality (Visnjic et al., 2018), which ultimately results in severe physical and mental health problems or even the disease (Haug et al., 2015). Meanwhile, smartphone addiction could also bring a number of psychological pressures to college students (Jun, 2016; Wang et al., 2017; Ou-Yang et al., 2022; Kao, 2023). Therefore, how to efficiently decreasing smartphone addiction has become a huge and pressing challenge for us now. According to the results from the current work, physical activity could significantly and negatively predict smartphone addiction, which means the higher level of physical activity the college students hold, the lower trend they would be addicted to smartphones, which is consistent with preceding research (Yang et al., 2021, 2022). Simultaneously, the theory of temporal self-regulation of physical activity (Salmon, 2001; Chen et al., 2022) suggests that regularly participating in physical activity will be conducive to personal health. Given that, as a stress-relieving entertainment activity, physical activity is not only beneficial for improving the physical fitness of college students, but also an effective method to decrease smartphone addiction for them. Consequently, it can be easily seen that regularly participating in different kinds of physical activity would be greatly beneficial for college students to decrease the passive effects from smartphone addiction.

Consistent with the findings from previous research (Sung, 2014; He et al., 2019), the present study found a negative correlation between self-esteem and smartphone addiction, and self-esteem could inversely and significantly predict smartphone addiction, indicating that improving college students’ self-esteem could effectively ameliorate their addiction to smartphones. In the light of the cognitive behavioral model about the Internet addiction, the non-adaptive cognitive and psychopathological factors of individuals can be viewed as the principal reasons for generating smartphone addiction, and the low-level self-esteem belongs to non-adaptive cognition (Davis, 2001). Quinones and Kakabadse (2015) revealed that individuals with low-level self-esteem could receive more positive evaluations and satisfying social experiences from others when engaging in online communication and other activities through their mobile phones. As we all know, as a virtual communication tool, Internet media might provide opportunities for those with low self-esteem to satisfy themselves and also shape themselves at the same time (Li et al., 2019), which inevitably makes them more dependent on using mobile phones for online communication to meet the demands they cannot achieve in the real life. Based on these mentioned above, it is not difficult to discover that college students with low self-esteem would be more likely to develop smartphone addiction, so the improvement of self-esteem would be an appropriate manner to solve this troublesome issue in coming days.

In addition, the present study also found that physical activity was significantly and positively correlated with the self-esteem. No doubt, as a feasible and effective exercise means, college students’ self-efficacy and physical self-esteem could be enhanced via physical activity, and then their overall self-esteem could be naturally improved. At the same time, different exercise methods, exercise intensity, exercise duration, and exercise frequency all have beneficial effect on college students, especially for those with low-level self-esteem (Ye, 2007). According to the physical self-perception profile (Fox and Corbin, 1989), individuals’ physical self-worth could be enhanced by the intervention with exercise, and then their athletic ability, physical condition, physical attractiveness, and strength would be also improved during that process, which ultimately leads to a remarkable increase in personal self-esteem. Furthermore, several scholars also proposed the notion that physical activity may improve individual’s self-esteem level, and then their sense of happiness in life would be also increased in a large degree (Eather et al., 2016; Sun and Zhang, 2020). Therefore, it can be easily seen that college students actively participating in physical activity during their daily life could possess a higher self-esteem than those lack of physical exercise.

Last but not least, the main finding of the current study was that self-esteem mediated the relationship between physical activity and smartphone addiction, which means that physical activity could improve addictive college students’ self-esteem and then indirectly decrease their smartphone addiction. According to the published research, people with lower self-esteem would be more likely to be dissatisfied with their lives compared to their counterparts (Hu et al., 2023), so that they usually attempt to compensate themselves by using electronic devices, especially the mobile phones, which would significantly increase the risk of smartphone addiction (Yang et al., 2004; He et al., 2020). However, a few previous studies have found that actively and regularly engaging in physical activity can not only alleviate academic pressure for college students (Zhang, 2016), but also get a healthier mentality (Xu et al., 2020), and such benefits as the improvement of personal self-esteem (Yìğìter, 2014; Zhang et al., 2017). Thus, personal smartphone addiction would be largely decreased, which mainly benefits from active participation in physical exercise in their daily lifestyle. According to the I-PACE model (Brand et al., 2016; Li et al., 2022), as a triggering factor for smartphone addiction, the lower level of physical activity will lead to the decrease of self-esteem, and then other abnormal methods would be used to compensate this deficiency for college students, such as using mobile phones to obtain personal satisfaction. And unfortunately, smartphone addiction would be finally hold by them. Based on these evidence above, it can reasonably draw a conclusion that self-esteem mediates the relationship between physical activity and smartphone addiction.

According to the pathway model for problematic use of the mobile phone summarized and suggested by Billieux (2012), he clearly points out that except for demographic variables, predictive factors in psychology for problematic mobile phone usage may contain two aspects, namely personality traits and its related psychological mechanisms, as well as self-esteem and its related psychological mechanisms, which means self-esteem would be an indispensable psychological variable to significantly predict individuals’ smartphone dependency. And then, based on this model mentioned above and the update evidence about this issue, Billieux et al. (2015) put forward a comprehensive model for problematic mobile phone use again, and also conclude three potential paths to predict problematic mobile phone use; among them, the excessive reassurance path reveals that low self-esteem will be a vital risky factor to effectively predict problematic mobile phone use. Therefore, it can be easily seen that self-esteem can be viewed as an essential factor to predict smartphone addiction; that is, the lower self-esteem you will have, the deeper smartphone addiction you will possess. Undoubtedly, the result from the current work also fairly demonstrate this standpoint. However, how to effectively improve personal self-esteem level so as to prevent and even ameliorate smartphone addiction in daily life? Fortunately, Mcauley et al. (2000) conduct a randomized controlled trial to examine the relationship between physical activity and self-esteem in older adults, and finally disclose that the self-esteem level of them display considerable enhancement after different forms of exercise intervention with 6 months. In addition, apart from the elderly, a systematic reviews about exercise to improve self-esteem in children and young people aged from 3 to 20 years also reveal that physical exercise may has positive short-term effects on self-esteem in children and young people (Ekeland et al., 2005). Obviously, this finding is consistent with the result from this research, and more important, the present study also enhance the external validity of this result, namely physical exercise could improve individuals’ self-esteem not only in children, young people, older adults, but also in college students aged over 20 years.

Nowadays, it is well known for us that as one of the most noticeable phenotype correlated with behavioral addictions all over the world, smartphone addiction has caused a large number of undesirable effects, as well as potential physical and mental harms to college students in daily life (Wang et al., 2017; Rathakrishnan et al., 2021; Ou-Yang et al., 2022; Kao, 2023). Therefore, how to properly deal with this puzzle by some efficient and reasonable manners has been put on agenda for relevant researchers, and it is fortunate that actively engaging and keeping in physical exercise would be a persuasive and practicable means to resolve this problem (Kim et al., 2015; Yang et al., 2019; Lin et al., 2022). Recently, a systematic review and meta-analysis presents that exercise would be an alternative approach for treating smartphone addiction, and this desirable intervention effect may be more significant in closed motor skills than in open motor skills, along with longer intervention duration may bring greater invention effects (Liu et al., 2019). Likewise, Kim (2013) also suggests that physical exercise would be beneficial to address this troublesome problem for people from both physiological and psychological aspects. And given that, the present research has explored the potential mediating mechanism between physical activity and smartphone addiction, and then also proved that self-esteem mediates the relationship between physical activity and smartphone addiction. Undoubtedly, this finding not only expands the related research in the field of behavioral addiction, but also serves as a feasible method for college students from China or even around the world to effectively resolve this troublesome issue in near future. That is to say, college students could not only indirectly improve their self-esteem by physical activity in daily life and then reduce their addiction to mobile phones, but also embrace this positive result through directly participating in various sorts of physical exercise.

## 5 Limitations

Despite those valuable implications discussed above, it is admittedly that there are also certain limitations existed in this study. Firstly, the present work conducted a cross-sectional survey research, thus the causal relationship can not be drawn by us. Secondly, the internal consistency coefficient of PARS-3 was only 0.693, and the reason is probably that this scale only has three items. Thirdly, the participants in this study were all from Guangzhou, which has limited the application and generalization of relevant results in some extent. Fourthly, even though self-esteem could mediate the relationship between smartphone dependency and physical activity, the indirect effect value seems somewhat small, and only accounting for 24% of the total effect. Given that, more related and potential variables need to be introduced to further explore the latent mediating role between physical activity and smartphone dependency. Fifthly, several advanced intelligent devices such as 3D-sensor pedometer and sports bracelet should be fully applied in near future, so that more accurate and reliable data could be naturally obtained by us, as well as more convincing and persuasive research consequences. In addition, it is necessary to mention that regarding assessment of duration with smartphone usage and personal subjective perception of its impact on individuals’ daily lives should be also taken into account, which is highly pivotal for understanding and recognizing smartphone addiction to us. Last but not least, although this study revealed the association between physical activity and smartphone dependency can be mediated by self-esteem, but in near future, more cross-sectional or longitudinal research, and even the related experimental designs should be conducted to further prove the reliability and validity of this result from the present study.

## 6 Conclusion

The present study intends to examine the relationship among physical activity, self-esteem, and smartphone dependency, especially whether the self-esteem plays a mediating role between physical activity and smartphone dependency, and eventually concludes that self-esteem could mediate the relationship between physical activity and smartphone dependency, indicating that college students could improve their self-esteem through participating in physical activity, and then their trend toward smartphone dependency could be largely decreased. Consequently, it can be easily seen that this highly reliable and practicable way of actively engaging in physical activity would be a best choose for college students in China to effectively get rid of smartphone dependency and other similar issues about addictive behavior, and then also help them develop a healthy behavior in daily life in coming days. However, it is admittedly that this result from the current work should be further testified through other longitudinal or cross-sectional research, and even the experimental studies so as to demonstrate its validity and reliability once again.

## Data availability statement

The original contributions presented in this study are included in this article/supplementary material, further inquiries can be directed to the corresponding authors.

## Ethics statement

The studies involving human participants were reviewed and approved by the Ethics Committee of South China University of Technology, and with the 1964 Declaration of Helsinki and its later amendments or comparable ethical standards. And the participants all provided their written informed consent before formally engaging in this study.

## Author contributions

YK: Data curation, Formal analysis, Investigation, Writing – original draft. XL: Conceptualization, Formal analysis, Methodology, Validation, Writing – review & editing. XX: Data curation, Formal analysis, Investigation, Project administration, Writing – original draft. BH: Data curation, Formal analysis, Investigation, Writing – original draft. JW: Data curation, Formal analysis, Investigation, Writing – review & editing. LZ: Data curation, Formal analysis, Investigation, Validation, Writing – review & editing. HW: Conceptualization, Data curation, Formal analysis, Investigation, Methodology, Project administration, Writing – review & editing. GY: Conceptualization, Formal analysis, Funding acquisition, Investigation, Methodology, Project administration, Validation, Writing – review & editing.
